# Lowered Risk of Nasopharyngeal Carcinoma and Intake of Plant Vitamin, Fresh Fish, Green Tea and Coffee: A Case-Control Study in Taiwan

**DOI:** 10.1371/journal.pone.0041779

**Published:** 2012-07-27

**Authors:** Wan-Lun Hsu, Wen-Harn Pan, Yin-Chu Chien, Kelly J. Yu, Yu-Juen Cheng, Jen-Yang Chen, Mei-Ying Liu, Mow-Ming Hsu, Pei-Jen Lou, I-How Chen, Czau-Siung Yang, Allan Hildesheim, Chien-Jen Chen

**Affiliations:** 1 Genomics Research Center, Academia Sinica, Taipei, Taiwan; 2 Institute of Biomedical Sciences, Academia Sinica, Taipei, Taiwan; 3 Division of Preventive Medicine and Health Service Research, National Health Research Institutes, Miaoli, Taiwan; 4 Molecular and Genomic Epidemiology Research Center, China Medical University Hospital, Taichung, Taiwan; 5 Division of Cancer Prevention, National Cancer Institute, National Institutes of Health (NIH), Department of Health and Human Services (DHHS), Bethesda, Maryland, United States of America; 6 Graduate Institute of Epidemiology and Preventive Medicine, College of Public Health, National Taiwan University, Taipei, Taiwan; 7 National Institute of Cancer Research, National Health Research Institutes, Chunan, Taiwan; 8 Graduate Institute of Microbiology, College of Medicine, National Taiwan University, Taipei, Taiwan; 9 Center of General Education, National Taipei University of Nursing and Health Sciences, Taipei, Taiwan; 10 Department of Otolaryngology, National Taiwan University Hospital and National Taiwan University College of Medicine, Taipei, Taiwan; 11 Department of Otorhinolaryngology, Head and Neck Surgery, Chang Gung Memorial Hospital and Chang Gung University, Taoyuan, Taiwan; 12 Division of Cancer Epidemiology and Genetics, National Cancer Institute, National Institutes of Health (NIH), Department of Health and Human Services (DHHS), Bethesda, Maryland, United States of America; Van Andel Institute, United States of America

## Abstract

**Background:**

A case-control study was conducted to evaluate the role of adult diet on nasopharyngeal carcinoma (NPC) in Taiwan.

**Methods:**

A total of 375 incident NPC cases and 327 controls matched to the cases on sex, age, and residence were recruited between July 1991 and December 1994. A structured questionnaire inquiring complete dietary history, socio-demographic characteristics, and other potential confounding factors was used in the personal interview. Unconditional logistic regression analysis was used to estimate multivariate-adjusted odds ratio (OR_adj_) with 95% confidence interval (CI) after accounting for known risk factors.

**Results:**

Fresh fish (OR_adj_, 0.56; 95% CI, 0.38–0.83 for the highest vs. lowest tertile of intake), green tea (OR_adj_, 0.61; 95% CI, 0.40–0.91 for drinking ≥1 times/week vs. never) and coffee (OR_adj_, 0.56; 95% CI, 0.37–0.85 for drinking ≥0.5 times/week vs. never) were inversely associated with the NPC risk. No association with NPC risk was observed for the intake of meats, salted fish, fresh vegetables, fruits and milk. Intake of vitamin A from plant sources was associated with a decreased NPC risk (OR_adj_, 0.62; 95% CI, 0.41–0.94 for the highest vs. lowest tertile).

**Conclusion:**

The study findings suggest that certain adult dietary patterns might protect against the development of NPC.

## Introduction

Nasopharyngeal carcinoma (NPC) is a rare cancer in most countries around the world with an incidence rate generally less than 1 per 100,000 person-years. However, the NPC incidence is extremely high in Southern China (25–30 per 100,000 person-years) [1], [2]. In Taiwan, an intermediate-risk area, the annual incidence rates for males and females in 2007 were 8.41 and 2.93 per 100,000 person-years, respectively [3].

Infection with Epstein-Barr virus (EBV) is considered a necessary cause of NPC. Long-term cigarette smoking, occupational exposures such as formaldehyde and wood dust, and genetic factors have also been documented as risk factors for NPC [4]–[8]. The dietary factors have been hypothesized to be involved in the development of NPC, but the evidence remains unclear except for Cantonese salted fish consumption in early childhood. Ho first proposed that salted fish consumption might be a risk factor for NPC [9]. In subsequent studies, intake of salted fish in childhood and adulthood was found to be associated with an excess risk of NPC in high-risk areas such as Guandong, Guangxi, and Hong Kong [10]–[13]. Even among individuals seropositive for immunoglobulin A antibodies against viral capsid antigen of EBV (anti-EBV VCA IgA), salted fish consumption during adulthood was associated with a 2-fold increase in NPC risk for those who had the highest consumption compared with whom never consumed [12]. However, no significant association during childhood and adulthood was found in low-risk areas including Philippines, Singapore, and United States [14]–[16].

In addition to salted fish at young ages, the intake of preserved foods has been found to be an NPC risk factor in many populations. In a meta-analysis of six case-control studies on the associations between preserved vegetable consumption in adulthood and NPC risk, the pooled odds ratio (95% confidence interval [CI]) was 2.04 (1.43–2.92) for the highest intake of preserved vegetables compared to the lowest intake [17]. In our previous study, the nitrosamine and nitrite consumption in childhood was significantly associated with an increased NPC risk [18].

In addition to salted fish and preserved foods, several studies reported an inverse relationship between consumption of vegetables and fruits and risk of NPC [16], [17]. The highest intake of fresh vegetables intake was associated with a 36% decrease in the risk of NPC in a meta-analysis [17]. A large case-control study conducted in China also reported a decrease NPC risk associated with the consumption of herbal tea and slowly cooked herbal soups [13].

We have reported the associations with NPC for various food items previously, with an emphasis on early life exposures [18]. While consumption of salted fish could not be fully evaluated due to the rarity with which this food item was consumed in our population, we did observe a positive association between early life consumption of non-soy foods high in nitrate/nitrosamines and risk of NPC. With respect to adult diet, we previously reported (17) that individuals in the highest quartile of intake of salted eggs or hot chili were at a significantly elevated risk of NPC compared to the lowest quartile. No significant association with NPC was observed for the intake of fresh soybean products, cured meats, smoked meats, fermented soybean products, preserved vegetables and fruits. However, this previous analysis did not evaluate macronutrient consumption and did not adjust for effects of potential confounders. In present analysis, we examined the associations between a wide range of food groups and macronutrients and NPC risk after taking known risk factors for NPC into consideration.

## Materials and Methods

### Study subjects

Details of this case-control study have been described previously [19], [20]. Briefly, incident cases of histologically confirmed NPC and matched community controls were enrolled between July 15, 1991 and December 31, 1994. NPC cases were restricted to individuals less than 75 years old, no previous diagnosis for NPC and residence in Taipei city/county for more than 6 months. One control was selected for each case recruited, individually-matched on sex, age (within 5-years), and residence area (same district or township). In total, there are 378 cases and 372 controls identified. Of these, risk factor questionnaires were obtained from 375 (99%) cases and 327 (88%) controls. Institutional Review Boards at the National Taiwan University in Taiwan and the National Cancer Institute in The United States approved the study protocol and informed consent. Written consent was obtained from study participants.

### Data collection

Participants were interviewed by trained nurses using a structured questionnaire. Information on socio-demographic characteristics, cigarette smoking, betel quid chewing, alcohol consumption, residential history, medical history, occupational history, as well as adult and childhood dietary history was collected. Complete food consumption was assessed with a food frequency questionnaire (FFQ) including 66 food items in most common Taiwanese diet. The information collected on dietary intake was the dietary history 3–10 years before ascertainment (biopsy date for cases and date at contact for controls). Participants were asked to indicate their average intake frequency per day, per week, per month, per year or less than once per year. For the present study, we investigated 3 food groups: 1) meat, fish, seafood and egg; 2) vegetables and fruits; and 3) milk, soybean milk, fresh fruit juice, tea and coffee.

### EBV seromarkers testing

Peripheral blood samples were collected from 369 cases and 320 controls. Serum was taken at that time of enrollment and stored at −80°C until assay. The sera were tested for various anti-EBV antibodies including viral capsid antigen (VCA) IgA, EBV nuclear antigen 1 (EBNA1) IgA, early antigen (EA) IgA, DNA binding protein IgG, and anti-DNase [21]–[24]. Individuals positive for any one EBV seromarker were classified as seropositive, and those negative to all seromarkers as seronegative. Totally, there were 358 cases and 97 controls seropositive for anti-EBV markers.

### Statistical analysis

Total calories and macronutrient intake were estimated using the Taiwan food composition database by multiplying the intake frequency for each food item by the nutrient content for a standardized portion size [25], [26]. The intake of various dietary items and macronutrients was categorized into three groups based on the tertiles in controls except some food items with an extreme intake frequency.

Unconditional logistic regression analyses were used to assess the multivariate-adjusted odds ratio (OR_adj_) and its corresponding 95% confidence interval (CI). All OR_adj_ were adjusted for age, gender, ethnicity, educational level, NPC family history, total calories, years of cigarette smoking, and exposures to formaldehyde and wood dust. Further stratification analyses were carried out to estimate OR_adj_ for individuals seropositive for anti-EBV markers. The dose-response relationship between NPC risk and various dietary factors was tested for statistical significance of the trend using an ordinal variable in the model. The correlations between intake of food items and macronutrient were assessed by Spearman correlation coefficients. All statistical tests were two-tailed.

## Results

A total of 371 NPC cases and 321 unaffected controls were included in the analyses. The male proportion was 69.5% and 69.2% for cases and controls, respectively. The mean age (standard deviation) was 45.6 (11.6) years for cases and 46.0 (11.7) years for controls. Compared with controls, the cases were more likely to be Fukienese in ethnicity. The educational level was lower and the proportion with a family history of NPC was higher in cases than controls.

Table 1 shows the consumption frequency of meat, egg, and seafood in NPC cases and controls. No significant association was observed between NPC risk and intakes of meat and egg. Compared to the reference group of taking fresh fish of ≤2 times/week, the OR_adj_ was 0.92 (95% CI, 0.61–1.40) and 0.56 (95% CI, 0.38–0.83) for those with the fresh fish intake of 2–6 and >6 times/week, respectively (p for trend<0.01) after adjustment for age, gender, ethnicity, educational level, NPC family history, total calories intake, years of cigarette smoking, and exposures to formaldehyde and wood dust. The OR_adj_ for the fresh fish intake remained similar in the analysis restricted to cases and controls seropositive for anti-EBV markers. No significant associations with NPC risk were observed for the intake of Cantonese-style salted fish (OR_adj_, 0.88; 95% CI, 0.35–2.21), although our ability to evaluate this association was limited by the small number of individuals reporting consumption of this food item. The intake of other seafood was also not significantly associated with NPC.

**Table 1:** Meat, egg and seafood consumption frequencies of 371 nasopharyngeal carcinoma cases and 321 community controls.

Variables (times/week)	Cases (%)	Controls (%)	Adjusted odds ratio (95% confidence interval)+	
			All subjects	Subjects seropositive for any anti-EBV
Lean pork and lean beef				
≤3.5	159 (42.9)	144 (45.3)	1.00 (referent)	1.00 (referent)
3.5–7	164 (44.2)	125 (39.3)	1.29 (0.91–1.82)	1.15 (0.70–1.91)
>7	48 (12.9)	49 (15.4)	1.15 (0.69–1.92)	1.71 (0.74–3.95)
P for trend			0.31	0.23
Fatty meat				
≤0.3	120 (32.6)	119 (37.2)	1.00 (referent)	1.00 (referent)
0.3–2	124 (33.7)	102 (31.9)	1.27 (0.86–1.88)	1.61 (0.90–2.89)
>2	124 (33.7)	99 (30.9)	1.20 (0.81–1.79)	1.21 (0.67–2.20)
P for trend			0.35	0.45
Chicken, duck and goose				
≤0.6	129 (34.9)	111 (34.6)	1.00 (referent)	1.00 (referent)
0.6–1.5	128 (34.6)	110 (34.3)	0.91 (0.62–1.35)	1.04 (0.58–1.88)
>1.5	113 (30.5)	100 (31.2)	1.02 (0.68–1.55)	0.95 (0.51–1.79)
P for trend			0.93	0.89
Salted, smoked and barbecued meat				
≤0.25	137 (38.1)	103 (33.0)	1.00 (referent)	1.00 (referent)
0.2–0.7	108 (30.0)	105 (33.7)	0.83 (0.56–1.24)	1.07 (0.58–1.97)
>0.7	115 (31.9)	104 (33.3)	0.89 (0.59–1.35)	0.79 (0.44–1.41)
P for trend			0.58	0.42
Egg				
≤2	129 (35.0)	108 (33.6)	1.00 (referent)	1.00 (referent)
2–6	128 (34.7)	100 (31.2)	1.09 (0.73–1.61)	0.84 (0.46–1.54)
>6	112 (30.4)	113 (35.2)	0.85 (0.56–1.29)	0.58 (0.31–1.09)
P for trend			0.45	0.09
Fresh fish				
≤2	131 (35.3)	93 (29.1)	1.00 (referent)	1.00 (referent)
2–6	109 (29.4)	83 (25.9)	0.92 (0.61–1.40)	0.75 (0.40–1.41)
>6	131 (35.3)	144 (45.0)	0.56 (0.38–0.83)**	0.48 (0.27–0.87)*
P for trend			<0.01	0.01
Cantonese-style salted fish				
No	362 (97.6)	306 (95.6)	1.00 (referent)	1.00 (referent)
Yes	9 (2.4)	14 (4.4)	0.88 (0.35–2.21)	4.80 (0.55–42.3)
Other seafood				
≤0.5	108 (29.4)	101 (31.7)	1.00 (referent)	1.00 (referent)
0.5–3	136 (37.1)	108 (33.9)	1.28 (0.86–1.91)	1.17 (0.64–2.13)
>3	123 (33.5)	110 (34.5)	1.02 (0.67–1.54)	0.85 (0.46–1.56)
P for trend			0.99	0.55

+ Adjusted for age, gender, ethnicity, educational level, NPC family history, total calories intake, years of cigarette smoking, and exposures to formaldehyde and wood dust.

Sample size in categories varies by available data.

** p<0.01.

* p<0.05.

The associations with NPC risk for the intake of vegetable and fruit are shown in Table 2. There was a weak negative association between NPC risk and intakes of all fresh vegetables (p for trend, 0.05). The intake of dark green vegetables was inversely associated with NPC risk after adjustment for other risk factors. Compared to the lowest intake, OR_adj_ for the highest intake was 0.65 (0.41–1.02) for all fresh vegetables and 0.67 (0.43–1.04) for dark green vegetables. No association with NPC risk was observed for the consumption of carrots, bean pods, gourds, preserved vegetables, fruit, and oranges/tangerines. Similar results were observed in analyses restricted to individuals seropositive for anti-EBV.

**Table 2:** Vegetable and fruit consumption frequencies of 371 nasopharyngeal carcinoma cases and 321 community controls.

Variables (times/week)	Cases (%)	Controls (%)	Adjusted odds ratio (95% confidence interval)+	
			All subjects	Subjects seropositive for any anti-EBV
Fresh vegetable				
<14	98 (26.4)	71 (22.1)	1.00 (referent)	1.00 (referent)
14–21	179 (48.3)	145 (45.2)	0.87 (0.58–1.30)	0.77 (0.42–1.43)
≥21	94 (25.3)	105 (32.7)	0.65 (0.41–1.02)	0.59 (0.30–1.16)
P for trend			0.05	0.12
Dark green vegetable				
<7	91 (24.6)	53 (16.5)	1.00 (referent)	1.00 (referent)
7–14	148 (40.0)	134 (41.7)	0.71 (0.46–1.09)	0.58 (0.29–1.18)
≥14	131 (35.4)	134 (41.7)	0.67 (0.43–1.04)	0.51 (0.25–1.04)
P for trend			0.10	0.08
Carrots				
≤0.3	151 (41.0)	121 (37.8)	1.00 (referent)	1.00 (referent)
0.3–1	106 (28.8)	93 (29.1)	0.94 (0.64–1.39)	0.91 (0.51–1.63)
≥1	111 (30.2)	106 (33.1)	0.84 (0.57–1.24)	0.82 (0.46–1.47)
P for trend			0.38	0.51
Bean pods				
≤0.5	152 (41.1)	114 (35.6)	1.00 (referent)	1.00 (referent)
0.5–1	108 (29.2)	94 (29.4)	0.91 (0.61–1.34)	0.88 (0.49–1.56)
>1	110 (29.7)	112 (35.0)	0.80 (0.55–1.18)	0.92 (0.52–1.65)
P for trend			0.26	0.77
Gourds				
<1	137 (37.1)	95 (29.7)	1.00 (referent)	1.00 (referent)
1–1.5	131 (35.5)	117 (36.6)	0.83 (0.56–1.21)	0.76 (0.42–1.35)
≥1.5	101 (27.4)	108 (33.8)	0.73 (0.49–1.10)	0.68 (0.37–1.26)
P for trend			0.13	0.21
Preserved vegetable				
≤0.04	130 (35.4)	112 (35.1)	1.00 (referent)	1.00 (referent)
0.04–0.4	119 (32.4)	101 (31.7)	1.05 (0.71–1.55)	1.16 (0.66–2.05)
<0.4	118 (32.2)	106 (33.2)	1.00 (0.67–1.48)	1.23 (0.68–2.23)
P for trend			1.00	0.49
Fresh fruit				
<3	133 (35.9)	82 (25.6)	1.00 (referent)	1.00 (referent)
3–7	182 (49.1)	180 (56.1)	0.77 (0.53–1.12)	0.69 (0.39–1.22)
>7	56 (15.1)	59 (18.4)	0.80 (0.48–1.32)	0.62 (0.29–1.30)
P for trend			0.28	0.17
Oranges/Tangerines				
≤2	138 (37.2)	111 (34.6)	1.00 (referent)	1.00 (referent)
2–6	94 (25.3)	79 (24.6)	1.06 (0.69–1.61)	0.97 (0.51–1.82)
>6	139 (37.5)	131 (40.8)	1.03 (0.71–1.51)	1.02 (0.59–1.78)
P for trend			0.87	0.94

+ Adjusted for age, gender, ethnicity, educational level, NPC family history, total calories intake, years of cigarette smoking, and exposures to formaldehyde and wood dust.

Sample size in categories varies by available data.

No significant association with NPC was observed for the consumption of milk, soybean milk as shown in Table 3. The consumption of fresh fruit juices and black tea was borderline significant with NPC risk (P for trend = 0.05). Significant reverse associations were found for the increasing intake of oolong tea (OR_adj_, 0.66 for >3 vs. 0 time/week; 95% CI, 0.44–0.98) and green tea (OR_adj_, 0.61 for 1+ vs. 0 time/week; 95% CI, 0.40–0.91). A significant inverse trend in risk was found for drinking coffee (OR_adj_, 0.56 for 0.5+ vs. 0 time per week, 95% CI, 0.37–0.85; p for trend = 0.01). The reverse associations with NPC risk for green tea and coffee remained statistically significant in the analyses restricted to cases and controls seropositive for anti-EBV markers.

**Table 3:** Beverage consumption frequencies of 371 nasopharyngeal carcinoma cases and 321 community controls.

Variables (times/week)	Cases (%)	Controls (%)	Adjusted odds ratio(95% confidence interval)+	
			All subjects	Subjects seropositive for any anti-EBV
Milk				
≤0.05	142 (38.8)	107 (34.0)	1.00 (referent)	1.00 (referent)
0.05–2	136 (37.2)	101 (32.0)	1.10 (0.74–1.64)	0.88 (0.49–1.58)
>2	88 (24.0)	107 (34.0)	0.79 (0.52–1.20)	0.90 (0.48–1.69)
P for trend			0.27	0.74
Soybean milk				
≤0.3	139 (37.5)	120 (37.5)	1.00 (referent)	1.00 (referent)
0.3–1	94 (25.3)	92 (28.8)	0.84 (0.55–1.27)	0.63 (0.34–1.15)
>1	138 (37.2)	108 (33.8)	1.13 (0.78–1.65)	1.02 (0.58–1.80)
P for trend			0.55	0.97
Fresh fruit juices				
0	219 (59.0)	158 (49.4)	1.00 (referent)	1.00 (referent)
≤0.5	77 (20.8)	79 (24.7)	0.74 (0.49–1.10)	0.86 (0.47–1.59)
>0.5	75 (20.2)	83 (25.9)	0.69 (0.46–1.04)	0.76 (0.41–1.38)
P for trend			0.05	0.35
Green tea				
0	268 (72.8)	194 (61.2)	1.00 (referent)	1.00 (referent)
<1	36 (9.8)	45 (14.2)	0.58 (0.35–0.98)*	0.43 (0.21–0.90)*
≥1	64 (17.4)	78 (24.6)	0.61 (0.40–0.91)*	0.40 (0.22–0.71)**
P for trend			0.01	<0.01
Black tea				
0	263 (71.1)	193 (60.3)	1.00 (referent)	1.00 (referent)
<0.5	53 (14.3)	61 (19.1)	0.66 (0.43–1.03)	0.65 (0.35–1.22)
≥0.5	54 (14.6)	66 (20.6)	0.69 (0.44–1.08)	0.97 (0.49–1.94)
P for trend			0.05	0.63
Oolong tea				
0	152 (41.4)	100 (31.3)	1.00 (referent)	1.00 (referent)
1–3	108 (29.4)	113 (35.3)	0.70 (0.47–1.03)+	0.75 (0.43–1.32)
>3	107 (29.2)	107 (33.4)	0.66 (0.44–0.98)*	0.83 (0.46–1.50)
P for trend			0.04	0.50
Coffee				
0	203 (54.9)	135 (42.1)	1.00 (referent)	1.00 (referent)
<0.5	94 (25.4)	87 (27.1)	0.74 (0.50–1.10)	0.54 (0.30–0.97)*
≥0.5	73 (19.7)	99 (30.8)	0.56 (0.37–0.85)**	0.48 (0.26–0.89)*
P for trend			0.01	0.01

+ Adjusted for age, gender, ethnicity, educational level, NPC family history, total calories intake, years of cigarette smoking, and exposures to formaldehyde and wood dust.

Sample size in categories varies by available data.

** P<0.01.

* p<0.05.

The associations with NPC risk for daily intake of selected macronutrients are shown in Table 4. No significant association with NPC was found for the intake of fat, carbohydrate, vitamin C, tocopherol and sodium. Compared with individuals with lowest tertile of protein intake, individuals with highest tertile of protein intake had a lower risk of NPC (OR_adj_, 0.50; 95% CI, 0.29–0.86; p for trend<0.01). Intake of vitamin A, especially vitamin A from plant source, was associated with a decreased NPC risk (OR_adj_, 0.62 for highest tertile vs. lowest tertile; 95% CI, 0.41–0.94; p for trend = 0.02)

**Table 4:** Macronutrient consumption frequencies of 371 nasopharyngeal carcinoma cases and 321 community controls.

Variables (per day)	Cases (%)	Controls (%)	Adjusted odds ratio (95% confidence interval)+	
			All subjects	Subjects seropositive for any anti-EBV
Protein intake (g)				
< = 58.5	162 (44.6)	105 (33.2)	1.00 (referent)	1.00 (referent)
58.5–76.5	105 (28.9)	105 (33.2)	0.59 (0.39–0.90)*	0.43 (0.22–0.83)*
>76.5	96 (26.5)	106 (33.5)	0.50 (0.29–0.86)*	0.39 (0.15–1.02)
P for trend			<0.01	0.04
Fat intake (g)				
< = 69.2	132 (36.4)	105 (33.2)	1.00 (referent)	1.00 (referent)
69.2–95.2	120 (33.1)	105 (33.2)	0.91 (0.61–1.36)	1.12 (0.63–2.00)
>95.2	111 (30.6)	106 (33.5)	0.94 (0.58–1.54)	1.35 (0.65–2.80)
P for trend			0.78	0.43
Carbohydrate intake (g)				
< = 245	137 (37.7)	105 (33.2)	1.00 (referent)	1.00 (referent)
245–346	128 (35.3)	105 (33.2)	0.90 (0.59–1.36)	0.75 (0.40–1.42)
>346	98 (27.0)	106 (33.5)	0.61 (0.35–1.05)	0.54 (0.22–1.32)
P for trend			0.09	0.18
Vitamin A (IU)				
< = 12340	155 (42.7)	105 (33.2)	1.00 (referent)	1.00 (referent)
12340–17450	114 (31.4)	105 (33.2)	0.70 (0.48–1.03)	0.54 (0.30–0.97)*
>17450	94 (25.9)	106 (33.5)	0.65 (0.43–0.99)*	0.50 (0.27–0.95)*
P for trend			0.04	0.03
Vitamin A from animal source (IU)				
< = 310	136 (37.5)	105 (33.2)	1.00 (referent)	1.00 (referent)
310–670	124 (34.2)	105 (33.2)	0.99 (0.67–1.46)	1.11 (0.61–2.00)
>670	103 (28.4)	106 (33.5)	0.84 (0.55–1.27)	0.88 (0.47–1.64)
P for trend			0.41	0.70
Vitamin A from plant source (IU)				
< = 11670	156 (43.0)	105 (33.2)	1.00 (referent)	1.00 (referent)
11670–16810	116 (32.0)	105 (33.2)	0.73 (0.50–1.06)	0.68 (0.38–1.21)
>16810	91 (25.0)	106 (33.5)	0.62 (0.41–0.94)*	0.50 (0.27–0.94)*
P for trend			0.02	0.03
Vitamin C (mg)				
< = 217.15	149 (41.1)	105 (33.2)	1.00 (referent)	1.00 (referent)
217.15–321	121 (33.3)	105 (33.2)	0.83 (0.57–1.21)	0.64 (0.36–1.15)
>321	93 (25.6)	106 (33.5)	0.75 (0.50–1.13)	0.62 (0.32–1.18)
P for trend			0.16	0.13
Tocopherol intake (ug)				
< = 11385	141 (38.8)	105 (33.2)	1.00 (referent)	1.00 (referent)
11385–15920	126 (34.7)	105 (33.2)	0.94 (0.63–1.40)	1.05 (0.58–1.91)
>15920	96 (26.5)	106 (33.5)	0.72 (0.45–1.14)	0.77 (0.39–1.54)
P for trend			0.17	0.49
Sodium intake (mg)				
< = 850	131 (36.1)	105 (33.2)	1.00 (referent)	1.00 (referent)
850–1660	140 (38.6)	105 (33.2)	1.13 (0.77–1.66)	1.16 (0.64–2.08)
>1660	92 (25.3)	106 (33.5)	0.78 (0.49–1.22)	0.70 (0.36–1.37)
P for trend			0.33	0.36

+ Adjusted for age, gender, ethnicity, educational level, NPC family history, total calories intake, years of cigarette smoking, and exposures to formaldehyde and wood dust.

Sample size in categories varies by available data.

* p<0.05.

The correlation coefficients among intakes of various dietary factors are shown in the Table S1. There were significant correlations between consumption frequencies of green tea and oolong tea drinking (correlation coefficient [r], 0.69; p<0.01) and between dark green vegetable and vitamin A from plant source (r, 0.66; p<0.01). The protein intake was also significantly correlated with the consumption of fresh fish (r, 0.28; p<0.01), dark green vegetables (r, 0.22; p<0.01), and coffee (r, 0.27; p<0.01). In the final logistic regression analysis, only the intake of fresh fish, green tea, coffee, and vitamin A from plant source was included in the regression model as shown in Table 5. After adjustment for age, gender, ethnicity, educational level, family NPC history, cigarette smoking, and exposures to formaldehyde and wood dust; significant associations with NPC risk were observed for the intake of fresh fish, green tea, coffee, and vitamin A from plant source. The OR_adj_, remained similar when the analyses were restricted to individuals with anti-EBV seropositivity.

**Table 5:** Multivariate analysis of risk factors associated with nasopharyngeal carcinoma.

Dietary intake	All subjects		Anti-EBV-seropositive subjects	
	Odds ratio (95% confidence interval)+	P value	Odds ratio (95% confidence interval)+	P value
Fresh fish (times/wk)				
≤2	1.00 (referent)		1.00 (referent)	
2–6	0.97 (0.63–1.48)	0.87	0.80 (0.42–1.55)	0.51
>6	0.58 (0.39–0.86)	0.01	0.50 (0.27–0.92)	0.03
P for trend		0.01		0.02
Green tea (times/wk)				
0	1.00 (referent)		1.00 (referent)	
<1	0.55 (0.32–0.94)	0.03	0.44 (0.21–0.96)	0.04
≥1	0.66 (0.44–1.00)	0.05	0.43 (0.24–0.78)	0.01
P for trend		0.02		0.003
Coffee (times/wk)				
0	1.00 (referent)		1.00 (referent)	
<0.5	0.80 (0.53–1.19)	0.27	0.61 (0.33–1.11)	0.10
≥0.5	0.61 (0.40–0.94)	0.02	0.55 (0.29–1.04)	0.07
P for trend		0.02		0.05
Vitamin A from plant source (IU/day)				
T1 (< = 11670)	1.00 (referent)		1.00 (referent)	
T2 (11670–16810)	0.73 (0.49–1.08)	0.11	0.65 (0.36–1.18)	0.16
T3 (>16810)	0.65 (0.43–0.99)	0.05	0.57 (0.30–1.08)	0.08
P for trend		0.04		0.07

+ Adjusted for age, gender, ethnicity, educational level, NPC family history, total calories intake, years of cigarette smoking, and exposures to formaldehyde and wood dust.

Sample size in categories varies by available data.

## Discussion

In this case-control study aimed to evaluate the associations with NPC risk for comprehensive food items during adult life, we found a significant reverse dose-response relationship between NPC risk and increasing consumption frequency of fresh fish, green tea, and coffee consumption. Salted fish intake and preserved foods have been identified as risk factors for NPC in many populations, especially during the weaning and childhood periods [10]–[13], [27]–[29]. These foods contain *N*-nitroso compounds which are considered carcinogenic to human. However, the associations with NPC for salted fish and preserved vegetables during adult life were not observed in the present study. One possible explanation may be very few cases and controls ever consumed Cantonese salted fish. Another explanation may be only the salted fish consumed in early childhood rather than adulthood is associated with the NPC risk.

Several studies reported a reverse association between vegetable consumption and NPC [30]–[34]. In our previous report, there were significant reverse associations between NPC and intake of dark green vegetable [18]. In the present analysis, there was a protective effect of vegetable consumption on NPC, but the association was not statistically significant after adjustment for other risk factors.

In contrast to another study [14], a reverse association between fresh fish consumption and NPC risk was observed in this study. The reverse associations between cancers and fish consumption had been observed in many epidemiological studies [35], [36]. The potential mechanism for the protective effect may be due to the nutrients such as ω-3 fatty acids in fish. Both docosahexaenoic acid (DHA) and eicosapentaenoic acid (EPA) have been shown to inhibit cell growth or decrease the risk of progression through inflammatory pathways in animal and in vitro studies [37].

The present study showed a significant reverse association between green tea consumption and NPC. This is consistent with another case-control study conducted in southern China (OR, 0.62) [38]. The association between tea consumption and decreased NPC risk is biologically plausible. The possible protective effect of tea consumption on various cancers has been evaluated in recent years, especially for green tea. In most animal studies, the extracts of tea could inhibit the formation and development of tumors at different sites [39]. Epigallocatechin gallate, one of the isomers in catechin, is a major component of green tea. It has a potent anti-microbial activity against bacteria, fungi, and viruses. Epigallocatechin gallate at 50 µM was reported to completely block EBV infection-induced cytokine expression and the EBV-induced B lymphocyte transformation [40].

An interesting reverse association between NPC risk and coffee consumption was observed in the present study after adjustment for other risk factors. Coffee is abundant in antioxidants such as caffeinic acid and chlorogenic acid. The coffee diterpenes, cafestol and kahweal have been considered to reduce the genotoxicity of several carcinogens by modifying detoxification enzymes [41], [42]. Coffee extracts were found to inhibit the virus activities *in vitro* [43]. However, the protective effects of tea and coffee on human cancers have been inconclusive in epidemiological studies [44]. The reasons for the discrepancy may be due to the differences in types of tea and coffee, consumption habits in various populations, and/or the adjustment for confounders.

EBV is considered the most important risk factor for NPC. The reverse associations with NPC for the intake of fresh fish, green tea and coffees remained statistically significant in the analyses limited to individuals seropositive for anti-EBV markers. In other words, the protective effects were independent from anti-EBV seropositivity and unlikely to be mediated through their effects on EBV lytic replication and resultant anti-EBV antibody seroconversion. In previous studies, NPC risk might be more relevant to the diet in childhood than the diet in adulthood [11], [13], [18]. It is worthwhile to examine whether the protective effect on NPC of fresh fish, tea and coffee consumed in adulthood as observed in this study also exist for the dietary items consumed in childhood.

The etiology of NPC involves genetic susceptibility, EBV infection, environmental factors and gene-EBV-environment interaction [45]–[47]. Recent GWAS studies have shown evidence of the important role for genetic factors play in the development of NPC [48], [49]. The absorption and metabolism of dietary component could be influenced by genetic polymorphisms. Components from diet could alter the genetic expression through epigenetic mechanisms [50]. Genistein and other isoflavones from soy and tea polyphenol(-)-epigallocatechin-3-gallate have been documented to reactivate methylation-silenced genes in cancer cell lines and inhibit cancer growth [51], [52].

This study has strengths and limitations. First of strengths, the dietary questionnaire included very comprehensive Taiwan food items and major micronutrient estimated from the inquired food items. The other advantage of this study was the control of potential confounding effects of other risk factors. Conversely, case-control studies are likely to be influenced by recall bias because cases might be more likely to remember and report exposure to known or possible risk factors than controls. However, we interviewed all cases before their diagnosis was confirmed by biopsy. Other limitations include the low consumption frequency of some food items, especially Guangdong salted fish, and the lack of portion size information in the questionnaire interview. Chinese food is very diverse and most dishes have mixtures of vegetables and meat; it is thus difficult to quantify the intake of various food items. Nonetheless, such limitations are likely to lead to bias towards the null value. Under such a conservative circumstance, we still observed a protective effect on NPC for the intake of fresh fish, green tea and coffee. If confirmed, the protective dietary factors reported herein could be incorporated into efforts to reduce NPC risk through dietary changes during adult life.

## Supporting Information

Table S1 Correlation coefficients among consumption frequency of selected food items and macronutrient in 321 controls.
(DOCX)
