# Supplementary material for: Lowered Risk of Nasopharyngeal Carcinoma and Intake of Plant Vitamin, Fresh Fish, Green Tea and Coffee: A Case-Control Study in Taiwan
Source: PLoS One. 2012 Jul 27;7(7):e41779. doi: 10.1371/journal.pone.0041779 (PMC3407060; doi:10.1371/journal.pone.0041779)
Supplement: Table S1 — Correlation coefficients among consumption frequency of selected food items and macronutrient in 321 controls. (DOCX) [file pone.0041779.s001.docx]

Table S1. Correlation coefficients among consumption frequency of selected food items and macronutrient in 321 controls

|  | Fresh fish | Dark green vegetables | Green tea | Oolong tea | Coffee | Protein intake | Plant- source vitamin A intake |
| --- | --- | --- | --- | --- | --- | --- | --- |
| Fresh fish | 1.00 | 0.06 | -0.01 | -0.02 | 0.04 | 0.28** | 0.09 |
| Dark green vegetables |  | 1.00 | <0.01 | 0.01 | -0.03 | 0.22** | 0.66** |
| Green tea |  |  | 1.00 | 0.69** | -0.02 | -0.07 | 0.01 |
| Oolong tea |  |  |  | 1.00 | 0.03 | -0.05 | 0.03 |
| Coffee |  |  |  |  | 1.00 | 0.27** | 0.11* |
| Protein |  |  |  |  |  | 1.00 | 0.40** |
| Plant-source vitamin A intake |  |  |  |  |  |  | 1.00 |

** p<0.01 *p<0.05
